# Esophageal mucosal integrity improves after laparoscopic antireflux surgery in children with gastroesophageal reflux disease

**DOI:** 10.1007/s00464-016-5304-0

**Published:** 2016-11-01

**Authors:** Femke A. Mauritz, Nicolaas F. Rinsma, Ernest L. W. van Heurn, Cornelius E. J. Sloots, Peter D. Siersema, Roderick H. J. Houwen, David C. van der Zee, Ad A. M. Masclee, José M. Conchillo, Maud Y. A. Van Herwaarden-Lindeboom

**Affiliations:** 1 Department of Pediatric Surgery, Wilhelmina Children’s Hospital, University Medical Center Utrecht, Room: KE.04.140.5, PO Box 85090, 3508 AB Utrecht, The Netherlands; 2 Department of Pediatric Gastroenterology and Hepatology, Wilhelmina Children’s Hospital, University Medical Center Utrecht, Utrecht, The Netherlands; 3 Department of Gastroenterology and Hepatology, Maastricht University Medical Center, Maastricht, The Netherlands; 4 Department of Pediatric Surgery, Maastricht University Medical Center, Maastricht, The Netherlands; 5 Department of Pediatric Surgery, Erasmus MC - Sophia Children’s Hospital, Rotterdam, The Netherlands; 6 Department of Gastroenterology and Hepatology, University Medical Center Utrecht, Utrecht, The Netherlands

**Keywords:** Gastroesophageal reflux disease, Laparoscopic fundoplication, Baseline impedance, Pediatrics

## Abstract

**Background:**

Esophageal intraluminal baseline impedance reflects the conductivity of the esophageal mucosa and may be an instrument for in vivo evaluation of mucosal integrity in children with gastroesophageal reflux disease (GERD). Laparoscopic antireflux surgery (LARS) is a well-established treatment option for children with proton pump inhibitory (PPI) therapy resistant GERD. The effect of LARS in children on baseline impedance has not been studied in detail. The aim of this study was to evaluate the effect of LARS on baseline impedance in children with GERD.

**Methods:**

This is a prospective, multicenter, nationwide cohort study (*Dutch national trial registry*: *NTR2934*) including 25 patients [12 males, median age 6 (range 2–18) years] with PPI-resistant GERD scheduled to undergo LARS. Twenty-four hour multichannel intraluminal impedance pH monitoring (MII-pH monitoring) was performed before and 3 months after LARS. Baseline impedance was evaluated during consecutive 2-h intervals in the 24-h tracings.

**Results:**

LARS reduced acid exposure time from 8.5 % (6.0–16.2 %) to 0.8 % (0.2–2.8 %), *p* < 0.001. Distal baseline impedance increased after LARS from 2445 Ω (1147–3277 Ω) to 3792 Ω (3087–4700 Ω), *p* < 0.001. Preoperative baseline impedance strongly correlated with acid exposure time (*r* −0.76, *p* < 0.001); however, no association between symptomatic outcome and baseline impedance was identified.

**Conclusions:**

LARS significantly increased baseline impedance likely reflecting recovery of mucosal integrity. As the change in baseline impedance was not associated with the clinical outcome of LARS, other factors besides mucosal integrity may contribute to symptom perception in children with GERD.

Multichannel intraluminal impedance (MII) combined with pH monitoring is a well-established technique for the assessment of gastroesophageal reflux disease (GERD) in both children and adults [1–4]. Changes in conductivity between multiple electrode pairs on a single-sensor MII-catheter allow detection of intra-esophageal movement of saliva during swallowing and the occurrence of gastroesophageal reflux (GER). In the absence of GER or swallowing, the esophagus is collapsed and the esophageal wall comes directly in contact with the MII-pH sensor catheter [5]. The impedance value during these periods, commonly referred to as baseline impedance, reflects the intrinsic electrical conductivity of the esophageal wall and may offer an in vivo tool to assess the integrity of the esophageal mucosa [6]. Baseline impedance values in healthy volunteers are usually high, whereas GERD patients express low baseline impedance values. Low baseline impedance values have been associated with Barrett’s esophagus or severe esophagitis [7], but recently they were also linked to acid-induced mucosal changes in patients with non-erosive reflux disease [6, 8, 9].

Furthermore, comparison with the golden standard, the Ussing Chamber technique showed that baseline impedance was closely correlated to TEER and fluorescein permeability as measured with the Ussing chamber [10].

Recovery of impaired mucosal integrity, reflected by increased baseline impedance, may relieve symptoms in GERD patients and be a marker for the clinical outcome of therapy [6, 11]. Previous studies evaluating the effect of proton pump inhibitors (PPIs) on baseline impedance in children and adults with GERD showed an increase in baseline impedance during acid suppressive therapy [11–13]. However, these studies did not reveal a correlation between the recovery of baseline impedance and symptomatic outcome [12, 13].

Laparoscopic antireflux surgery (LARS) to treat severe, PPI-therapy resistant GERD aims to reduce reflux episodes and symptoms [14]. At present, the effect of LARS on esophageal mucosal integrity has not previously been studied in detail. The aim of this study is to assess the effect of LARS on baseline impedance and to explore if changes in baseline impedance are associated with the clinical outcome of LARS.

## Methods

### Study design

We performed a prospective multicenter study in three University Medical Centers in the Netherlands that are specialized in performing fundoplication in children [Wilhelmina children’s Hospital, University Medical Center Utrecht (UMCU); Sophia’s Children’s Hospital, Erasmus University Medical Center (Erasmus MC) and Maastricht University Medical Center (MUMC)]. From July 2011 until December 2013, we prospectively included pediatric patients diagnosed with PPI-therapy resistant GERD. All patients had been treated with high dosages of PPI for at least 6 months, and GERD was defined as: (1) troublesome GERD symptoms, (2) pathological reflux on 24-h pH monitoring and (3) a positive SAP (symptom association probability) assessment (≥95 %).

Patients who underwent previous esophageal or gastric surgery (except previous gastrostomy placement) and those with structural abnormalities other than esophageal hiatal hernia were excluded. Patients were studied before and 3 months after the surgical procedure.

### Surgical procedure

All laparoscopic fundoplication procedures were performed by pediatric surgeons experienced in minimal invasive pediatric surgery. In the UMCU, the anterior, partial fundoplication according to Thal [15] was used to perform fundoplication. The other two centers (Erasmus MC and MUMC) used the posterior, total fundoplication according to Nissen [16]. Before fundoplication, the distal esophagus was fully mobilized, and the distal 3 cm of the esophagus was repositioned back into the abdomen. Both vagal nerves were identified, and a crusplasty was performed routinely (UMCU and EMC). Thereafter, the fundoplication was constructed. The Thal fundoplication was performed by plicating the fundus of the stomach over 270° against the distal anterior intra-abdominal part of the esophagus and the diaphragmatic crus [17]. A floppy Nissen was constructed with one of the sutures of the 360° posterior wrap incorporated in the esophageal wall [16].

### Ambulatory 24-h multichannel intraluminal impedance pH monitoring

Ambulatory 24-h MII-pH testing was conducted after at least 3-day cessation of all medications that affect gastrointestinal motility and/or acid secretion. Measurements were performed using a combined pH-impedance catheter assembly that consisted of six impedance segments and one ISFET pH electrode (Unisensor AG, Attikon, Switzerland). The pH electrode was positioned 5 cm above the upper border of the manometrically localized lower esophageal sphincter (LES). Impedance and pH signals were stored on a digital data logger (Ohmega, Medical Measurement Systems, Enschede, The Netherlands), using a sampling frequency of 50 Hz. Patients and/or their parents were instructed to continue their regular diet, to report GERD symptoms and to keep a diary of their consumptions and body position (supine or upright) during the measurement.

### Reflux-specific questionnaires

To assess reflux symptoms, patients and/or their parents were asked to fill out the validated age-adjusted Gastroesophageal Reflux Symptom Questionnaire (GSQ) before and 3 months after LARS [18]. Reflux symptoms and dysphagia were scored for frequency and severity on a score ranging from 1 (none) to 7 (most severe). Symptoms were defined as: no symptoms (no symptoms reported); mild (mild symptoms, weekly); moderate (mild symptoms, daily or severe symptoms, weekly) and severe (severe symptoms, daily). Reflux symptoms were scored using the symptoms heartburn, regurgitation, food refusal and vomiting.

### Data analysis

Baseline impedance values were calculated for two specific segments in the esophagus during consecutive 2-h intervals in the 24-MII-pH tracings as previously described [11]. Periods of ≥30 s not containing any swallows or gastroesophageal reflux episodes were selected, and the averaged impedance value over this specific time period at two of the in total six impedance segments (Z6-distal and Z2-proximal) was calculated, using a specific function incorporated in the analysis software (Ohmega, MMS, Enschede, The Netherlands). The 2-hourly obtained baseline impedance values for each segment were averaged and used for further analysis. The 24-h MII-pH tracings were further analyzed for acid exposure time, the number and acidity of reflux episodes according to previously described definitions [19]. Acid exposure time was defined as pathological when pH <4 during >6.0 % of time during 24-h monitoring [20, 21]. Reflux episodes reaching the proximal (z2) impedance segment were classified as proximal. Baseline impedance throughout the manuscript refers to baseline impedance in the distal (z6) segment, unless stated otherwise.

### Ethical approval and trial registration

This study was registered with the Dutch national trial registry (www.trialregister.nl; Identifier: 2934). Ethical approval for this prospective multicenter study was obtained from the University Medical Center Utrecht Ethics Committee, and local approval was obtained by the two participating centers (Erasmus MC and MUMC). Prior to initiating any trial-related study procedure, informed consent from the patients’ parents was obtained.

### Statistical analysis

Continuous parametric variables were expressed as mean ± standard error of the mean (SEM). Nonparametric variables were expressed as median, with interquartile ranges (IQR). For continuous parametric outcomes, a paired sample *T* test was performed. Nonparametric continuous outcomes were analyzed using the Wilcoxon signed-rank test. Correlations between different parameters were calculated using the Pearson correlation coefficient or Spearman’s rank correlation coefficient as appropriate. Linear regression analysis was performed to identify possible determinants of the effect of LARS on baseline impedance. Determinants of interest included: age, type of fundoplication and changes in acid exposure time. A *p* value below 0.05 was considered statistically significant. All statistical analyses were performed using commercially available computer software (IBM SPSS Statistics for Windows, Version 22. Armonk, NY: IBM Corp.).

## Results

In total, 25 children were included in our study. Mean age of the included patients was 6 (range 2–18) years at the time of fundoplication. Five children (80 %) had normal neurodevelopment (NN), while impaired neurodevelopment (NI) was seen in five children (20 %). Patient demographics are shown in Table 1. Thal fundoplication was performed in 18 and Nissen fundoplication in seven children. In all patients, fundoplication could be completed by laparoscopy.

**Table 1:** Baseline characteristics

	(Median; IQR)
Age at time of operation (years)	6.0 (3.0–11.0)
Duration of hospital admission (days)	3.0 (2.0–4.5)

	*n* (%)
Male gender	12 (48.0 %)
Impaired neurodevelopment	5 (20.0 %)
Gastrostomy preoperatively in situ	4 (16.0 %)

The caregivers of all 25 patients filled out both pre- and postoperative reflux symptom questionnaires. Pre-operative 24-h pH-MII tracings were completed in all 25 patients. After surgery, 24-h pH-MII could not be performed in two patients due to refusal of the tracing by the caregivers. Postoperative tracings were successfully completed in 23 of the 25 children. Preoperative esophagogastroduodenoscopy was performed in 18 patients. In 13 of these patients, macroscopic and/or microscopic acid-induced changes in the esophagus were observed.

### Baseline impedance before antireflux surgery

**Fig. 1:**
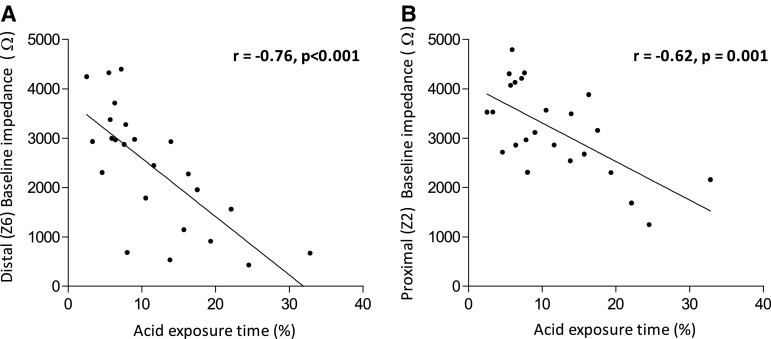
Correlation between esophageal acid exposure time (%) and **A** distal baseline impedance (Ω) and **B** proximal baseline impedance (Ω) in GERD patients before LARS

Median baseline impedance before surgery was 2245 Ω, with a range from 430 to 4401 Ω. Spearman’s rho correlation coefficient showed a strong negative correlation between distal baseline impedance and acid exposure time (*r* −0.76, *p* < 0.001) before surgery (Fig. 1A). Baseline impedance also negatively correlated with other reflux parameters, such as reflux episodes lasting longer than 5 min (*r* −0.55, *p* = 0.005) and number of acid reflux episodes (*r* −0.55, *p* = 0.005). The number of non-acid reflux episodes showed no correlation with baseline impedance (*r* −0.03, NS).

In patients who underwent preoperative endoscopy, a trend toward significance was observed when comparing baseline impedance in patients with macroscopic and/or microscopic signs of esophagitis (*n* = 13) to those with no sign of inflammation (*n* = 5) [resp. 1788 (IQR 677–3187 Ω) versus 2928 Ω (IQR 2591–4364 Ω), *p* = 0.09]. Baseline impedance showed no correlation with severity of reflux symptoms [severe symptoms 2376 Ω (IQR 1251–3275 Ω) versus mild/moderate symptoms 2925 Ω (IQR 1083–3207 Ω), NS].

Proximal baseline impedance was significantly higher when compared to distal baseline impedance [3116 Ω (IQR 2539–4071 Ω) versus 2445 Ω (IQR 1147–3277 Ω), *p* < 0.05]. Baseline impedance in the proximal segment also showed a negative correlation with acid exposure time (*r* −0.62, *p* = 0.001; Fig. 1B) and number of acid reflux episodes (*r* −0.50, *p* = 0.01).

Proximal extension of reflux episodes occurred on average in 44.8 % (SEM 4.8 %) of all reflux episodes. The number of reflux episodes reaching the proximal esophagus did not correlate with proximal baseline impedance (*r* −0.18, NS).

### Effects of laparoscopic antireflux surgery

**Fig. 2:**
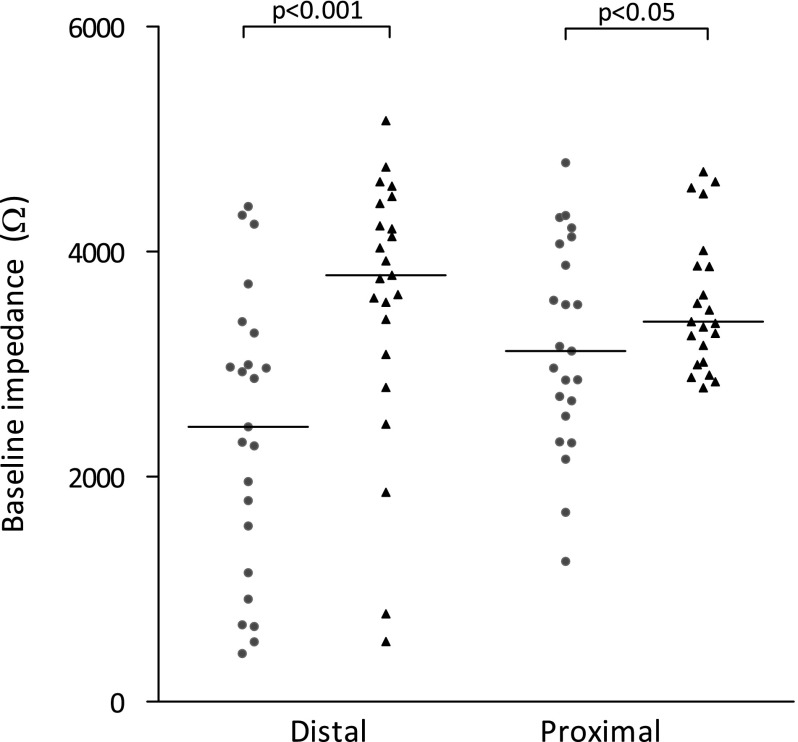
Baseline impedance in distal (z6) and proximal (z2) esophageal segments before (*red*) and after (*blue*) LARS

**Fig. 3:**
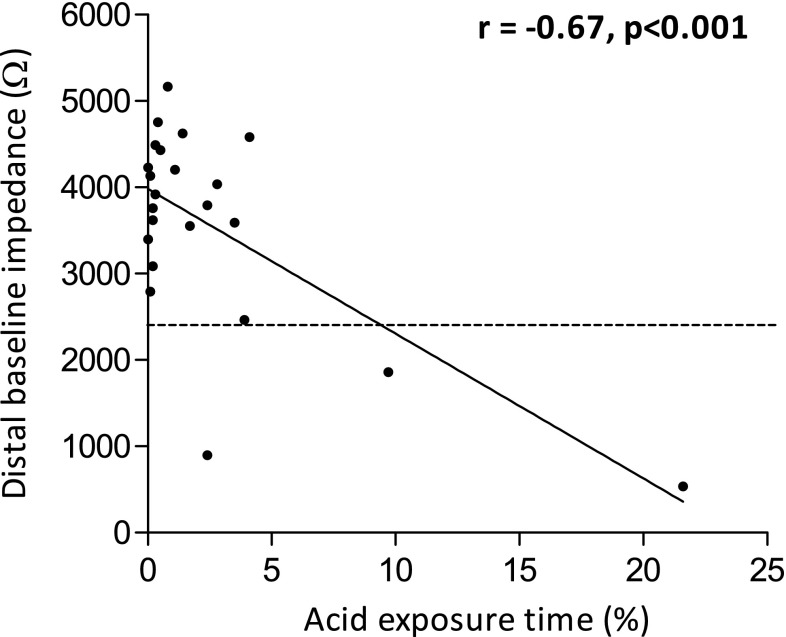
Correlation between acid exposure time (%) distal baseline impedance (Ω) and after LARS. The *horizontal dashed line* indicates preoperative median baseline impedance

LARS reduced acid exposure time from 8.5 % (6.0–16.2 %) to 0.8 % (0.2–2.8 %), *p* < 0.001, at 3-month follow-up. All other reflux parameters, including the number of proximal and non-acid GER episodes on 24-h MII-pH monitoring, were also significantly reduced after LARS (Table 2). LARS significantly increased baseline impedance in both the distal and proximal impedance segment (Fig. 2). Distal baseline impedance showed a significant correlation with remaining postoperative acid exposure time (*r* −0.67, *p* < 0.001) (Fig. 3). Furthermore, the change in distal baseline impedance after LARS correlated to the reduction in acid exposure time (*r* 0.48, *p* = 0.02).

**Table 2:** Reflux parameters on MII-pH monitoring

	Preoperative	Postoperative	*p* value
Acid exposure total (%, IQR)	8.5 (6.0–16.2)	0.8 (0.2–2.8)	<0.001
Upright (%, IQR)	12.1 (4.8–19.2)	1.8 (0.5–5.9)	0.001
Supine (%, IQR)	7.1 (0.9–15.3)	0 (0–0)	<0.001
Total GER (*n*, IQR)	92 (66–139)	14 (11–22)	<0.001
Acid GER (*n*, IQR)	61 (34–94)	8 (1–13)	<0.001
Non-acid GER (*n*, IQR)	23 (11–42)	5 (3–11)	<0.001
Proximal reflux (*n*, IQR)	36 (14–87)	1 (0–3)	<0.001

*GER* Gastroesophageal reflux, *n* number of reflux episodes, *IQR* interquartile range

The median preoperative baseline impedance was 2445 Ω. After LARS, the baseline impedance value in three patients was lower than this median preoperative baseline impedance value (Fig. 3). In two of these three patients, persisting pathologic acid exposure was found. The third patient with lower postoperative baseline impedance after the procedure had esophageal gastric metaplasia in the distal esophagus. Despite normalization of acid exposure, low baseline impedance persisted only in the most distal segment of the esophagus likely due to increased conductivity of gastric epithelium.

Overall reflux symptoms significantly decreased after LARS (*p* = 0.001). In 15 (65 %) patients, complete remission of reflux symptoms was achieved and in an additional 6 (26 %) patients symptom scores improved. In two of the 23 (9 %) patients, reflux symptoms were unaltered; one of these patients showed a low baseline value (536 Ω). Deterioration of reflux symptoms did not occur (Table 3). Four patients with postoperative reflux complaints and/or pathological reflux on 24-h pH monitoring still required PPI therapy.

**Table 3:** Symptom assessment

	Preoperative (*n*, %)	Postoperative (*n*, %)	*p* value
Reflux symptoms			
None	0 (0 %)	1 (65 %)	
Mild	2 (9 %)	5 (22 %)	0.001
Moderate	6 (26 %)	2 (9 %)	
Severe	15 (65 %)	1 (4 %)	

Similar to preoperative analysis, no association between postoperative symptoms and baseline impedance could be identified [no symptoms (*n* = 15) versus persistent symptoms (*n* = 8): 3917 Ω (IQR 3087–4490) versus 3706 Ω (2983–4373), NS].

### Determinants of the effect of LARS on baseline impedance

A linear regression analysis was performed to explore determinants of the effect of LARS on baseline impedance. Age (*β*: −3.5 Ω; 95 % CI −114.6; 107.7, *p* = 0.95) and type of fundoplication (*β*: −105.1 Ω; 95 % CI −1274.7; 1064.5, *p* = 0.95) did not affect the change in baseline impedance after LARS. Also, the change in acid exposure time (*β*: 50.7 Ω; 95 % CI −10.3; 111.7, *p* = 0.098) did not reach statistical significance.

## Discussion

In the present study, we investigated the effect of laparoscopic antireflux surgery (LARS) on baseline impedance as a reflection of mucosal integrity in pediatric patients with gastroesophageal reflux disease (GERD). Our main findings were that LARS resulted in recovery of baseline impedance, that reflux symptoms significantly decreased after LARS, without an association with an increase in baseline impedance and finally that no factors affecting the effect of LARS on baseline impedance could be identified.

LARS aims to prevent reflux events from the stomach thereby protecting the esophageal mucosa from potential stressors in the refluxate. Previous studies have shown a strong inverse correlation between baseline impedance and acid exposure in pediatric GERD patients [9, 12, 22]. Accordingly, we hypothesized that successful elimination of esophageal acid exposure by LARS would increase baseline impedance. Our current study confirms this hypothesis as it demonstrates a significant decrease in acid exposure time and an increase in baseline impedance, which likely reflects the recovery of the esophageal mucosa. Furthermore, postoperative low distal baseline impedance was observed in patients with persistent pathologic acid exposure time and in one patient with gastric metaplasia. In the latter patient, this could be due to the intrinsic higher conductive properties of columnar epithelium when compared to esophageal squamous epithelium [23].

Impaired mucosal integrity has been proposed as an important mechanism in symptom generation, as it allows acid reflux to permeate into the deeper layers of the mucosa and activate sensory nerve endings [24]. Previous studies have shown an association between esophageal mucosal integrity and acid sensitivity using a standardized acid perfusion test [25, 27]. Lower baseline impedance, as a marker of impaired mucosal integrity, has also been shown to correlate with clinical signs of GERD in the pediatric population [12]. Related to these findings, recovery of impaired mucosal integrity could hypothetically result in improvement of reflux symptoms in GERD patients [6]. Laparoscopic antireflux surgery showed good clinical outcome, but an association between GERD symptoms and baseline impedance despite the use of validated questionnaires could not be identified. These results are in accordance with previous studies attempting to identify a similar association after PPI treatment in children or after endoscopic fundoplication in adults [12, 13]. Together, these outcomes suggest that in addition to the mucosal integrity, also content, proximal extent and volume of the refluxate, as well as peripheral and central-mediated sensitivity, affect GERD symptom perception [26, 27].

Baseline impedance may allow identification of patients with impaired mucosal integrity after LARS, as it has been associated with esophagitis and microscopic changes of the mucosa [6, 9, 10, 28]. Post-procedural evaluation of GERD is important as patients with persistent GERD may be at risk of developing complications, such as esophagitis, stenosis, Barrett’s epithelium and ultimately adenocarcinoma of the esophagus [29]. GERD symptoms may not always be evident, especially in younger children or children with impaired neurodevelopment. Evaluation of mucosal impedance during ambulatory 24-h MII-pH testing may help to detect impaired mucosal integrity. MII-pH analysis is, however, a time-consuming and the procedure itself may be uncomfortable for pediatric patients. Recently, other groups have reported on endoscopy guided single-sensor catheters to measure mucosal impedance during endoscopy [30, 31]. These catheters enable instant evaluation of mucosal integrity during endoscopy instead of the normal 24-h MII-pH monitoring. Unfortunately, in children endoscopy is generally performed under general anesthesia. Because of the impact and risks of the anesthesia and the invasive way endoscopy is performed, this method in its current form seems unsuitable in pediatric patients. Future developments may lead to alternative methods that allow a similar instant evaluation of the esophageal mucosa without using endoscopy [32].

In the proximal esophagus, an inverse correlation between baseline impedance and (distal) acid exposure time was seen, whereas no correlation with the number of proximal reflux episodes was found. As the MII-pH catheter used in this study only had a distal pH sensor, it is not entirely clear whether proximal baseline impedance is directly affected by acid reflux reaching the proximal esophagus or by indirect mechanisms, activated by distal acid exposure [33]. Previously, Farre et al. [33] showed that distal acid perfusion resulted in changes in the mucosal integrity of the non-exposed proximal esophagus, which is suggestive for an indirect mucosal reaction spreading more proximally than the site of mucosal injury. Further insights in this possible mechanism of impaired mucosal integrity in the proximal esophagus may be of clinical importance as the proximal esophagus is often linked to symptom perception [34–36].

Determinants, such as age, type of fundoplication or change in acid exposure time, influencing the effect of LARS on baseline impedance could not be identified. Infants and young children previously were shown to have a lower baseline impedance when compared to older (pediatric) patients [37]. Age, however, did not influence the effect of LARS on baseline impedance. Until now, studies on efficacy of different types of fundoplication in both the adult and pediatric population were not able to show differences in reflux control [38, 39]. In this study type of fundoplication also was not a significant determinant for the effect of LARS on baseline impedance. The change in acid exposure time after LARS showed a tendency to influence the effect of LARS on baseline impedance and may have been a significant determinant, if more patients had been included.

The main limitation in this current study is the number of patients. A larger number of patients would have allowed us to investigate determinants of interest in a linear regression model with higher statistical power. Furthermore, in the current study endoscopy was not performed per protocol. In 18 out of 25 (72 %) patients, endoscopy was performed before LARS and in only a few of the patients after the procedure. Data to correlate the changes in baseline impedance with endoscopic and/or histological mucosal findings are therefore not available. Pardon et al. showed in adults that baseline impedance is closely correlated to evaluation of mucosal integrity by established ex vivo methodology (Ussing Chamber technique) [10]. Together with previous findings from an experimental model [6], these observations indicate that baseline impedance is a reliable tool for the evaluation of mucosal integrity in vivo.

In conclusion, LARS increased baseline impedance, which is likely to reflect the recovery of mucosal integrity. Although both baseline impedance and symptomatic reflux control increased, these two parameters were not mutually associated. Factors influencing the effect of LARS on baseline impedance could not be identified.

## Abbreviations

GER: Gastroesophageal reflux
GERD: Gastroesophageal reflux disease
GSQ: Gastroesophageal Reflux Symptom Questionnaire
LARS: Laparoscopic antireflux surgery
MII: Multichannel intraluminal impedance
PPI: Proton pump inhibitor
